# Dapagliflozin and short-term changes on circulating antigen carbohydrate 125 in heart failure with reduced ejection fraction

**DOI:** 10.1038/s41598-023-37491-5

**Published:** 2023-06-30

**Authors:** Martina Amiguet, Patricia Palau, Eloy Domínguez, Julia Seller, Jose Manuel Garcia Pinilla, Rafael de la Espriella, Gema Miñana, Alfonso Valle, Juan Sanchis, Jose Luis Górriz, Antoni Bayés-Genís, DAPA VO2 investigators, Eloy Domíngueza, Clara Sastre, Gema Miñana, Enrique Santas, Anna Mollar, Jose Civera, Adriana Conesa, Rim Zakarne, Ainoha Larumbe, Jose Manuel Garcia Pinilla, Juan Jose Gómez Doblas, Ainhoa Robles Mezcua, Gema Miñana, Vicent Bodí, Domingo Pascual-Figal, Clara Jiménez Rubio, Alejandro I. Pérez Cabeza, Arancha Díaz Expósito, José David Martínez Carmona, Manuel Luna Morales, Francisco J. Zafra Sánchez, Ángel Montiel Trujillo, Herminio Morillas Climent, Julio Núñez

**Affiliations:** 1 FISABIO, Universitat Jaume I, Castellón, Spain; 2 Cardiology Department, Servicio de Cardiología, Hospital Clínico Universitario de Valencia, Universitat de València, INCLIVA, Valencia, Spain; 3 Cardiology Department, Hospital de Denia, Alicante, Spain; 4 Cardiology Department, Hospital Universitario Virgen de la Victoria, IBIMA, Malaga, Spain; 5 CIBER Cardiovascular, Madrid, Spain; 6 Nephrology Department, Hospital Clínico Universitario de Valencia, Universitat de València, INCLIVA, Valencia, Spain; 7 Department and Heart Failure Unit, Hospital Universitari Germans Trias i Pujol, Badalona, Spain; 8 Universitat Autònoma de Barcelona, Barcelona, Spain; 9 Cardiology Department, Hospital Universitario Virgen de la Arrixaca, Murcia, Spain

**Keywords:** Biomarkers, Cardiology, Diseases

## Abstract

Circulating antigen carbohydrate 125 (CA125) has emerged as a proxy of fluid overload in heart failure. This study aimed to evaluate the effect of dapagliflozin on short-term CA125 levels in patients with stable heart failure with reduced ejection fraction (HFrEF) and whether these changes mediated the effects on peak oxygen consumption (peakVO_2_). This study is a post-hoc sub-analysis of a randomized, double-blinded clinical trial in which 90 stable patients with HFrEF were randomly assigned to receive either dapagliflozin or placebo to evaluate change in peakVO_2_ (NCT04197635). We used linear mixed regression analysis to compare changes in the natural logarithm of CA125 (logCA125) and percent changes from baseline (Δ%CA125). We used the “rwrmed” package to perform mediation analyses. CA125 was available in 87 patients (96.7%). LogCA125 significantly decreased in patients on treatment with dapagliflozin [1-month: Δ − 0.18, (CI 95% = − 0.33 to − 0.22) and 3-month: Δ − 0.23, (CI 95% = − 0.38 to − 0.07); omnibus p-value = 0.012]. Δ%CA125 decreased by 18.4% and 31.4% at 1 and 3-month, respectively (omnibus p-value = 0.026). Changes in logCA125 mediated the effect on peakVO_2_ by 20.4% at 1 month (p < 0.001). We did not find significant changes for natural logarithm of NTproBNP (logNT-proBNP) [1-month: Δ − 0.03, (CI 95% = − 0.23 to 0.17; p = 0.794), and 3-month: Δ 0.73, (CI 95% = − 0.13 to 0.28; p-value 0.489), omnibus p-value = 0.567]. In conclusion, in patients with stable HFrEF, dapagliflozin resulted in a significant reduction in CA125. Dapagliflozin was not associated with short-term changes in natriuretic peptides. These changes mediated the effects on peakVO_2._

## Introduction

Sodium-glucose cotransporter 2 inhibitors (SGLT2i) are the first-line therapy in patients with heart failure with reduced ejection fraction (HFrEF)^1^. However, the mechanisms underlying these benefits remain not fully understood. Decongestion effects have been proposed among potential drivers of these benefits^2^. Some studies suggest a differential decongestive effect of SGLT2i beyond traditional diuretic agents by a predominant reduction of extravascular congestion rather than intravascular space contraction^2,3^. Circulating levels of CA125 have emerged as a widely available proxy of tissue congestion and inflammation in heart failure (HF)^4,5^.

CA125 is upregulated in most patients with acute heart failure (AHF), and its values identify patients with greater fluid overload and a higher risk of adverse events^4^.

Interestingly, the trajectory of CA125 weeks after decompensation is strongly associated with the risk of adverse events^4^. A recent observational retrospective study suggested a significant reduction of CA125 in patients with type 2 diabetes and stable HFrEF following the initiation of empagliflozin^6^.

The DAPA-VO2 trial examined the effects of dapagliflozin in 90 stable patients with heart failure with reduced ejection fraction (HFrEF) in New York Heart Association Class (NYHA class) II or III. These patients were randomly assigned to receive either dapagliflozin or placebo. The primary outcome measured the change in peak oxygen consumption (peakVO2) at 1 and 3 months. Main results showed that patients treated with dapagliflozin experienced a significant increase in peakVO2 compared to the 27placebo group at both 1 and 3 months (1-month: + Δ 1.09 ml/kg/min, 95% confidence interval [CI] 0.14–2.04; p = 0.021, and 3-month: + Δ 1.06 ml/kg/min, 95% CI 0.07–2.04; p = 0.032)^7^.

In this substudy, we aimed to evaluate short-term changes in CA125 after dapagliflozin initiation in patients with HFrEF and whether these changes mediate the effect of dapagliflozin in maximal function capacity assessed by peak oxygen consumption (peakVO_2_). Additionally, we explored changes in the N-terminal pro-hormone of brain natriuretic peptide (NT-proBNP).

## Methods

### Study sample and procedures

This study was an investigator-initiated, multicenter, double-blind, randomized clinical trial designed to evaluate the effect of dapagliflozin on maximal functional capacity in patients with HFrEF. The patients were randomized 1:1 to receive either dapagliflozin or a placebo. Maximal functional capacity was evaluated at three time points: baseline, 1, and 3 months after initiation of treatment.

The study protocol was approved by Agencia Española del Medicamento y Productos sanitarios (AEMPS) and by Comité Ético de Investigación Clínica (CEIC) del Hospital Clínico Universitario de Valencia. This study protocol was previously registered at http://clinicaltrials.gov (NCT04197635, 01/11/2019) and published elsewhere^7^. All methods were performed in accordance with the relevant guidelines and regulations. All patients provided signed informed consent.

The study population included patients with stable chronic HF in NYHA II or III class, and left ventricular ejection fraction (LVEF) ≤ 40%. The eligibility criteria, study procedures, and main findings were published elsewhere^7^.

Briefly, randomized patients performed a baseline, 1 and 3-month maximal cardiopulmonary exercise test (CPET) using incremental and symptom-limited cardiopulmonary exercise testing on a bicycle ergometer, beginning with a workload of 10 W and gradually increasing in a ramp protocol of 10 W increments every 1 min.

Blood samples were obtained the same day before CPET. NT-proBNP and CA125 were measured using standard commercial enzyme immune analysis (Roche Elecsys® NT-proBNP and Roche Elecsys® CA 125 assays). The values of both biomarkers were blinded to those researchers in charge of performing the CPET.

### Statistical analysis

All statistical comparisons were made under a modified intention-to-treat principle. Continuous variables are expressed as means (± 1 SD) or medians (interquartile range [IQR]), and discrete variables are presented as percentages. At baseline, the means, medians, and frequencies among treatment groups were compared using the t-test, Wilcoxon test, and chi-square test, respectively. Different correlations between logarithm of CA125 (logCA125) and the logarithm of NT-proBNP (logNT-proBNP), and log-transformed exposures and peakVO2 were assessed by the Pearson test.

A linear mixed regression model (LMRM) was used to analyze changes in peakVO_2_. Because of hierarchical levels of nesting—the period within patients and patients within centers—the model included patient ID and study center as random intercepts. All analyses included the baseline value of the endpoint and the marker (exposure of interest) as covariates (ANCOVA framework). CA125 and NT-proBNP were transformed to their natural logarithm to make their distributions more symmetrical and closer to normal. Additionally, we evaluated percent changes from the baseline of both biomarkers (Δ%CA125 and Δ%NT-proBNP). The period effect was included by modelling the interaction between the treatment and the period. LMRM results are presented as least square means with 95% confidence intervals (CIs) and p-values.

We used the “rwrmed” package to perform mediation analysis using regression with residuals^8^, a method for decomposing an overall effect of treatment into direct and indirect components when treatment-induced confounding is present. We also explored logCA125 and logNT-proBNP changes across baseline values of both biomarkers (low vs. high using recognized prognostic cutpoints: 23 U/ml for CA125 and 1000 pg/ml for NT-proBNP)^9–11^. Additionally, we tested changes in logCA125 and logNT-proBNP as potential mediators of the effect of dapagliflozin on peakVO_2_. Because the period effect of the treatment was not homogeneous in the LMRM analyses, these markers were tested as mediators at 30 and 90-day independently. All mediation models included the baseline value of peakVO_2_ and the marker (mediator). The CA125 and NT-proBNP models included hemoglobin as a pre- and post-treatment covariate because it is a marker highly affected by dapagliflozin treatment^12^. The center effect was accounted for by including it as a cluster variable. To investigate the statistical stability of the results, a bootstrap re-sampling procedure was employed based on 300 bootstrap samples (sampling with replacement).

All analyses were performed with STATA 16.1 [Stata Statistical Software, Release 16 (2019); StataCorp LP, College Station, TX, USA].

## Results

Between May 2019 and October 2021, 90 patients were randomized. CA125 was available in 87 patients (96.7%) at baseline. The mean age of this study sample was 67.0 ± 10.5 years, 27 (36.0%) had type 2 diabetes, 48 (55.2%) showed prior ischemic heart disease, and 72 (82.8%) were on stable NYHA II. At baseline, the medians (p25%-p75%) of CA125 and NT-proBNP were 11 U/ml (7–17) and 1274 pg/ml (895–2267), respectively. The proportion of patients treated with sacubitril/valsartan, β-blockers, and mineralocorticoid receptor antagonists was 88.5%, 87.4%, and 74.7%, respectively. No significant differences in baseline characteristics across treatment arms were observed. (Table 1). We did not find correlations between baseline logCA125 and logNT-proBNP (r = 0.080; p = 0.461). At baseline, logNT-proBNP (r = − 0.460, p < 0.001) but not logCA125 (r = − 0.134, p = 0.216) was correlated with peakVO2.

**Table 1:** Baseline characteristics of the patients stratified by randomization arm.

Variables	All patients	Placebo	Dapagliflozin	p-value
n	87	42	45	
Demographic and medical history				
Age, years	69 (61–74.2)	67.5 (60.1–74.3)	69.8 (62.4–74)	0.816
Men, n (%)	66 (75.9)	31 (73.8)	35 (77.8)	0.666
BMI, kg/m^2^	27.8 ± 4.3	28.3 ± 4.3	27.3 ± 4.4	0.317
Hypertension, n (%)	67 (77)	34 (81)	33 (73.3)	0.399
Diabetes mellitus, n (%)	29 (32.2)	13 (28.9)	16 (35.6)	0.499
Dyslipidemia, n (%)	57 (65.5)	28 (66.7)	29 (64.4)	0.827
Current smoker, n (%)	19 (21.8)	10 (23.8)	9 (20)	0.667
Prior smoker, n (%)	27 (31)	15 (35.7)	12 (26.7)	0.362
NYHA II, overall, n (%)	78 (89.7)	37 (88.1)	41 (91.1)	0.644
Comorbidities				
Heart rate, b.p.m.	70 (61–82)	71 (63–83)	70 (60–80)	0.531
Systolic blood pressure, mmHg	118 (110–128)	117.5 (110–130)	120 (110–124)	0.972
Diastolic blood pressure, mmHg	60 (60–70)	60 (60–70)	60 (60–70)	0.813
Prior history of IHD, n (%)	48 (55.2)	21 (50)	27 (60)	0.349
Prior history of COPD, n (%)	23 (26.4)	13 (31)	10 (22.2)	0.356
Atrial fibrillation, (%)	49 (56.3)	23 (54.8)	26 (57.8)	0.777
Left bundle branch block, n (%)	14 (16.1)	9 (21.4)	5 (11.1)	0.524
Laboratory values				
Haemoglobin, g/dl	14.3 ± 1.7	14.3 ± 1.7	14.2 ± 1.8	0.809
eGFR, ml/min/1.73 m^2^	66.4 ± 21.8	68.8 ± 23	64.1 ± 20.7	0.309
Serum sodium, mEq/l	139.9 ± 2.5	140 ± 2.6	139.9 ± 2.5	0.837
NT-proBNP, pg/ml	1279.5 (885–2267)	1839 (924–2416)	1085 (889–1688)	0.297
CA125, U/ml	10.8 (7–16.1)	11 (7.5–17.5)	9.2 (6–16)	0.711
Echocardiography				
LVEF, %	35.4 (30.2–37.8)	35.4 (30–37.9)	35.4 (30.2–37.7)	0.925
Left atrial volume index, ml/m^2^	42 (30.9–51.7)	42 (33–56.7)	39.9 (30.1–51.7)	0.651
E/E′ ratio	12.8 (9–15.4)	13.3 (9.2–15)	12.2 (8.8–15.4)	0.969
CPET				
PeakVO_2_, ml/kg/min	13 ± 3.3	12.5 ± 3.1	13.4 ± 3.5	0.228
Percent predicted peakVO2,%	55.9 (48.8–67.3)	55.9 (48.8–65.7)	55.9 (49.7–67.9)	0.419
VE/VCO2slope	34.7 (32.3–39.)	36.3 (32.8–39.4)	33.8 (31.5–38.8)	0.845
RER	1.22 ± 0.13	1.20 ± 0.12	1.23 ± 0.13	0.349
Medical treatment				
Loop diuretics, n (%)	74 (85.1)	35 (83.3)	39 (86.7)	0.663
ACEI or ARB or Sacubitril-Valsartan, n (%)	84 (96.6)	40 (95.2)	44 (97.8)	0.517
Sacubitril-Valsartan, n (%)	77 (88.5)	37 (88.1)	40 (88.9)	0.908
MRA, n (%)	65 (74.7)	30 (71.4)	35 (77.8)	0.496
β-blockers, n (%)	76 (87.4)	35 (83.3)	41 (91.1)	0.275

Values are expressed by mean ± standard deviation. Categorical variables are presented as percentages.

Continuous variables are presented as median (interquartile range), and categorical variables are as percentages.

ACEI, angiotensin-converting enzyme inhibitor; ARB, angiotensin receptor blocker; BMI, body mass index; CA125, Antigen Carbohydrate 125; COPD, chronic obstructive pulmonary disease; CPET, cardiopulmonary exercise testing; eGFR, estimated glomerular filtration rate; IHD, ischemic heart disease; LVEF: left ventricular ejection fraction assessed by Simpson method; MRA, mineralocorticoid receptor antagonists; NYHA, New York Heart Association functional class; NT-proBNP, the natural logarithm of N-terminal prohormone of brain natriuretic peptide: PeakVO_2,_ peak oxygen uptake; RER, respiratory exchange ratio; VE/VCO2slope, ventilatory efficiency.

### Changes in CA125

**Figure 1:**
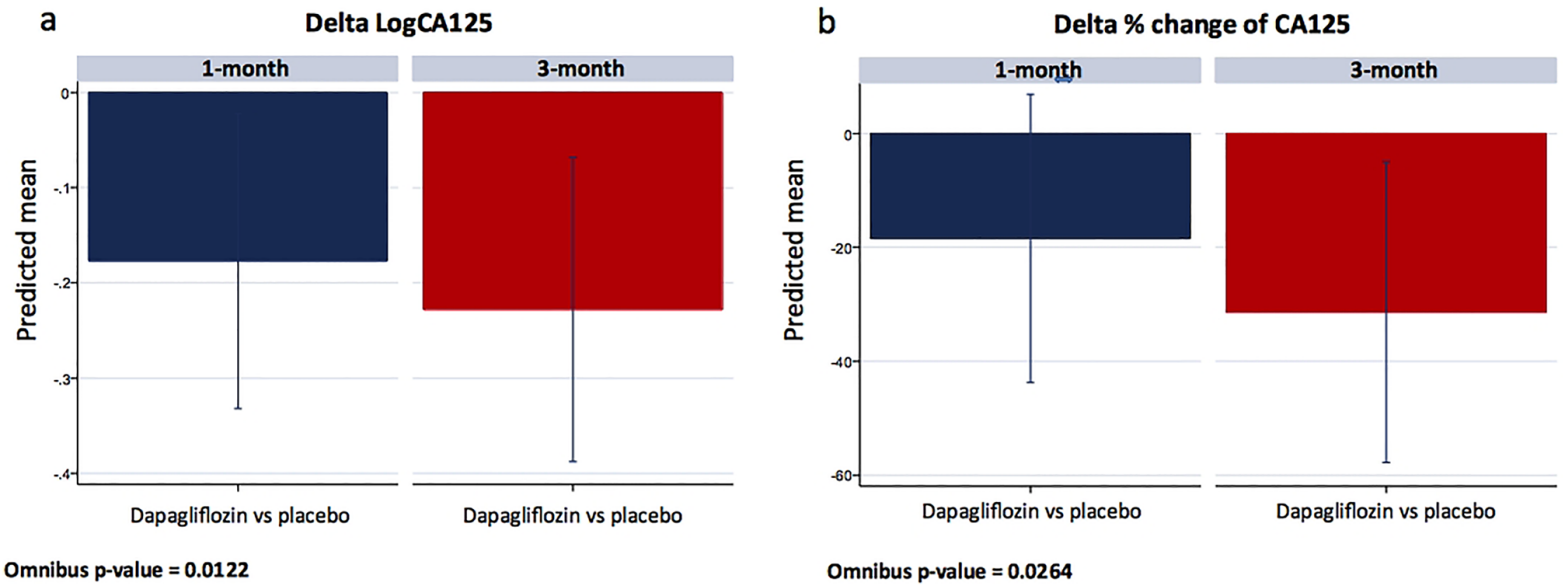
Changes in logCA125 (**a**) and percentage changes in CA125 (**b**) between dapagliflozin and placebo at 1-month and 3-month. CA125, antigen carbohydrate 125, logCA125, the natural logarithm of antigen carbohydrate 125.

LogCA125 significantly decreased in patients on treatment with dapagliflozin [1-month: Δ − 0.18, (CI 95% = − 0.33 to − 0.22; p = 0.025), and 3-month: Δ − 0.23, (CI 95% = − 0.38 to − 0.07; p-value = 0.005), omnibus p-value = 0.012] as is shown in Fig. 1a. When CA125 percent changes from baseline were evaluated, we found a significant decrease of 18.4% and 31.4% at 1 and 3-month, respectively (omnibus p-value = 0.026), as is shown in Fig. 1b.

After adjusting for post-treatment confounding by hemoglobin and pre-treatment values of peakVO_2_, mediation analysis showed that logCA125 significantly mediated the effect of dapagliflozin on peakVO_2_ by − 20.4% (p < 0.001) at 1-month. At 3-month, logCA125 changes did not mediate the changes in peakVO2 (− 9.3%, p = 0.453).

### Changes in NT-proBNP

**Figure 2:**
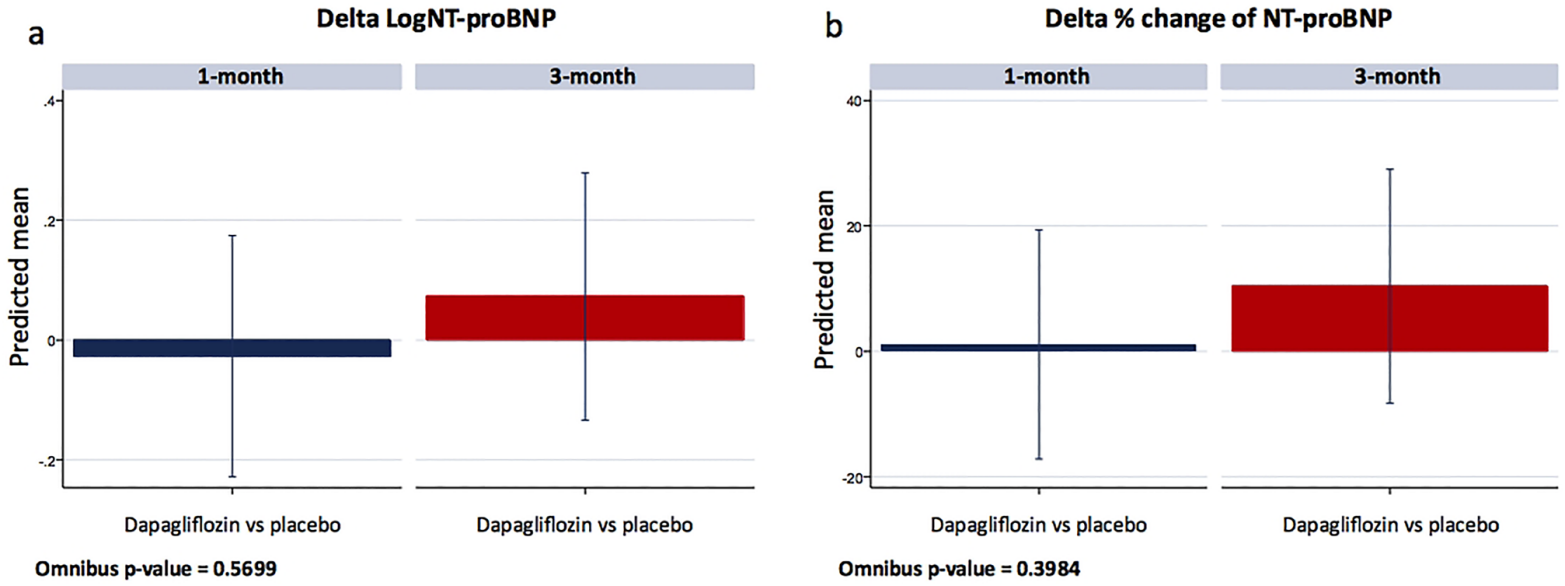
Changes in logNT-proBNP (**a**) and percentage changes in NT-proBNP (**b**) between dapagliflozin and placebo at 1-month and 3-month. LogNT-proBNP, the natural logarithm of N-terminal prohormone of brain natriuretic peptide; NT-proBNP, N-terminal prohormone of brain natriuretic peptide.

We did not find a significant between treatment changes for logNT-proBNP [1-month: Δ − 0.03, (CI 95% = − 0.23 to 0.17; p = 0.794), and 3-month: Δ 0.73, (CI 95% = − 0.13 to 0.28; p-value = 0.489), omnibus p-value = 0.567] (Fig. 2a).

When NTproBNP percent changes from baseline were evaluated, we did not find a significant between-treatment decrease [1-month: Δ 1.1%, (CI 95% = − 17.2 to − 19.3; p = 0.909), and 3-month: Δ 10.4%, (CI 95% = − 8.3 to 29.0; p-value = 0.275), omnibus p-value = 0.398] (Fig. 2b). Changes in logNT-proBNP did not mediate the changes of dapagliflozin in peakVO_2_ [− 0.49% (p = 0.311) and − 3.10% (p = 0.191) at 1 and 3-month, respectively].

### Changes in both biomarkers stratified across baseline values.

**Figure 3:**
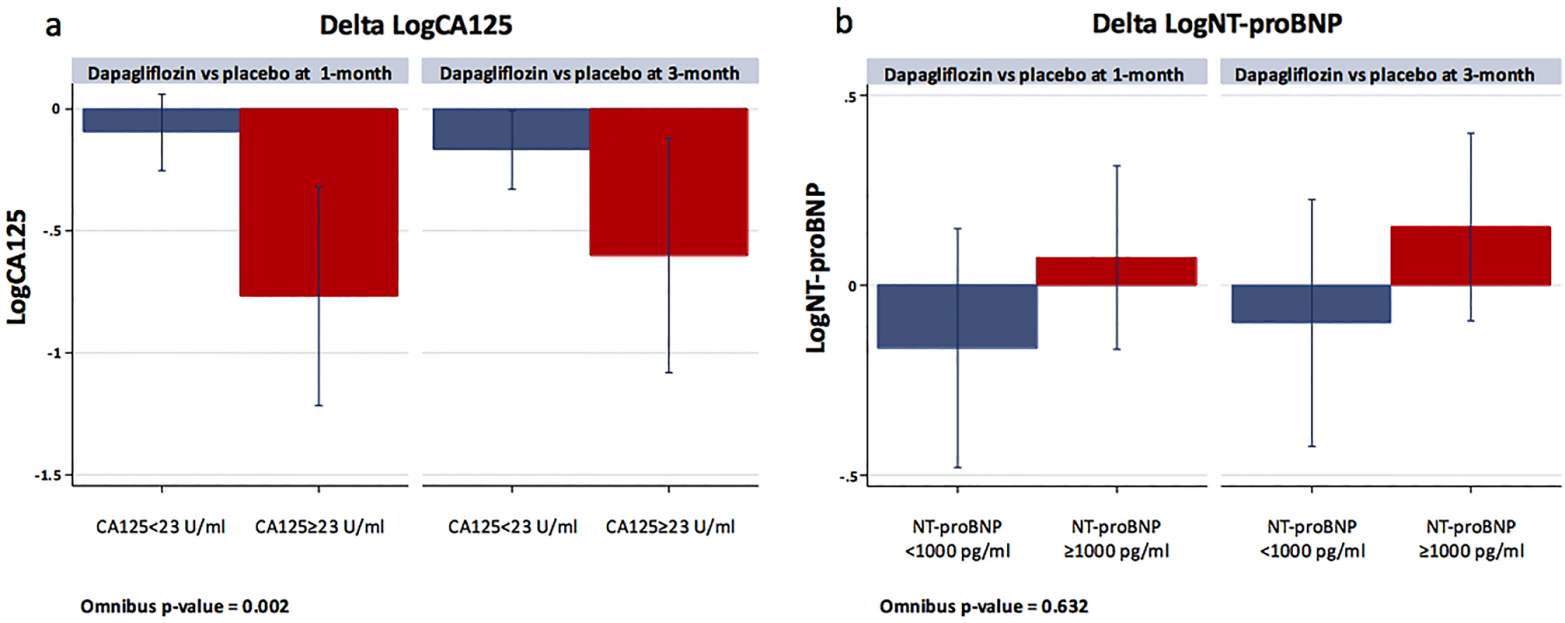
Changes in logCA125 (**a**) and logNT-proBNP (**b**) stratified across baseline values at 1-month and 3-month. CA125, antigen carbohydrate 125, logCA125, the natural logarithm of antigen carbohydrate 125; logNT-proBNP, the natural logarithm of N-terminal prohormone of brain natriuretic peptide; NT-proBNP, N-terminal prohormone of brain natriuretic peptide.

When changes in logCA125 were stratified across the baseline values of CA125 (< 23 vs. ≥ 23 U/ml), we identified a greater reduction of logCA125 in those with CA125 at baseline ≥ 23 U/ml (omnibus p-value between treatments = 0.002), as is shown in Fig. 3a. For logNT-proBNP, there was not a heterogeneous effect of dapagliflozin across the baseline cutoff (< 1000 and ≥ 1000 pg/ml). At both strata, between-treatment comparisons were not significant at both time points (Fig. 3b).

## Discussion

In this study that enrolled patients with stable HFrEF, dapagliflozin treatment was associated with a significant short-term reduction of circulating CA125 but not NT-proBNP. Furthermore, changes in CA125 were more remarkable in patients at higher values at baseline. Interestingly, changes in CA125 mediated the increase in peakVO_2_ found in this study.

The natriuresis and osmotic diuresis associated with SGLT2 inhibition have been shown in some studies to be associated with an increased plasma osmolarity and a modest decrease in plasma volume^2,3^. Despite being confirmed in dedicated studies, some authors argue that a preferential decrease in the interstitial volume leads to improved tissue perfusion and less risk of acute kidney injury with SGLT2i compared with loop and other diuretics^2^.

CA125, also known as mucin 16 (MUC16), is a complex glycoprotein primarily produced by mesothelial cells in the pericardium, pleura, or peritoneum in response to tissue congestion or inflammation^4,5^. Despite not being fully understood, the interaction between transmembrane mucins and adjacent proteins supports the involvement of CA125 in processes that regulate fluid and cell transport, including inflammation, tissue repair, and tumor dissemination^5^. Specifically, CA125 has been found to play a role in suppressing natural killer cell activity and acts as a ligand for mediators involved in reparative responses^4^. Currently, the clinical utility of CA125 as a biomarker is mainly associated with the evaluation of patients suspected or diagnosed with ovarian cancer.

However, CA125 is upregulated in two-thirds of patients with AHF syndrome regardless of left ventricular systolic function and is associated with clinical congestion, especially tissue and third-space fluid accumulation^4^. More interestingly, the fluctuation of this glycoprotein in the first weeks after decompensation is strongly associated with the risk of other adverse clinical events suggesting a promissory role in monitoring decongestion^4^. In a prior pre-post retrospective analysis that included 60 patients with type 2 diabetes and HFrEF, the authors found that empagliflozin prescription was related to a significant reduction of CA125^6^. In a more controlled scenario, the current findings confirm the prior observation and endorse prior assumptions indicating a predominant tissue decongestion effect (reduction of CA125) following the initiation of dapagliflozin. Although patients evaluated here were stable and with low values of CA125 at randomization, we could identify a significant decrease in CA125, and their changes mediated the functional improvement seen in this study (increase in peakVO_2_ by 8.4%).

Regarding changes in NT-proBNP, in the current study, we found a non-significant reduction of − 10.4% at 3-month in those allocated to dapagliflozin. It is consistent with previous results of the DAPA-HF and EMPEROR-reduced trial in which the authors reported a 8 and 12-month reduction of about 10%^13,14^.

Further studies in acute heart failure scenarios are warranted. We speculate that changes in CA125 in response to decongestive therapies, including SGLT2i and renewed drugs such as acetazolamide^15^, may be helpful as a proxy of decongestion, especially in patients admitted with AHF.

Interestingly, the greater magnitude of CA125 changes was found in those patients with higher values at baseline, suggesting this effect is mainly found in those with residual fluid overload. Similar findings, in terms of greater reduction in those with higher baseline values, were also reported by our group in daily clinical practice^6^. Thus, we speculate that changes in CA125 after initiation of dapagliflozin may capture information about changes in subclinical/residual congestion. Along this line of thought, recent observations suggest that the optimal cutpoint of CA125 for risk stratification in acute HF is lower than the classically cutoff used in cancer studies^4,9^.

Beyond emerging as a reliable parameter of tissue congestion, some other advantages, such as low-cost, wide availability, and circulating levels not influenced by renal function and left ventricular ejection fraction, deserve to be highlighted^4,16,17^.

Further studies are required to unravel the exact mechanisms behind these findings. For instance, CA125 is also a proxy of inflammation. Thus, the effect attributable to dapagliflozin may also be capturing information about the antiinflammatory properties of this agent. Lastly, further trials are warranted to explore CA125 changes induced by SGLT2i in patients with greater fluid overload.

### Study limitations

Several limitations need to be acknowledged. First, this is a post-hoc analysis of a randomized clinical trial. Second, this study has the inherent limitations of being a trial with a relatively small number of participants with limited power. Third, we have exclusively evaluated stable HFrEF patients; thus, we cannot extrapolate these findings to other clinical scenarios. Finally, although the reduction of CA125 following the initiation of dapagliflozin may be attributed to the tissue decongestion and anti-inflammatory properties of SGLT2i, the current study did not include routine measurements of inflammatory biomarkers or clinical congestion scores. As a result, the precise biological mechanisms underlying the reduction of CA125 following dapagliflozin initiation were not elucidated in this study.

## Conclusions

In patients with stable HFrEF, dapagliflozin led to a significant short-term reduction of CA125 but not NT-proBNP. Changes in CA125 mediated the effect on the short-term maximal functional capacity. Further studies are warranted to confirm these findings in this and other HF clinical scenarios.

## Data Availability

Data are available on reasonable request to corresponding author. All data relevant to the study are included in the article.

## Abbreviations

CA125: Circulating antygen carbohydrate 125
HFrEF: Heart Failure with reduced ejection fraction
PeakVO_2_: Peak oxygen consumption
LogCA125: Logarithm of CA125
LogNTproBNP: Logarithm of NTproBNP
SGLT2i: Sodium glucose transporter type 2 inhibitor
HF: Heart Failure
AHF: Acute Heart Failure
NYHA: New York Heart Association
LMRM: Linear mixed regression model
CI: Confidence interval
